# Supplementary material for: Capability, Opportunity, and Motivation—Identifying Constructs for Increasing Physical Activity Behaviours in Women with Polycystic Ovary Syndrome (PCOS)
Source: Int J Environ Res Public Health. 2023 Jan 28;20(3):2309. doi: 10.3390/ijerph20032309 (PMC9915314; doi:10.3390/ijerph20032309)
Supplement: Supplementary file 1 [file ijerph-20-02309-s001.zip › ijerph-2110915-supplementary.pdf]

1. I have the PHYSICAL opportunity to *do enough physical activity for improvement to my health*

**What is PHYSICAL opportunity?**<br>The environment provides the opportunity to engage in the activity concerned.<br>(e.g., sufficient time, the necessary materials, reminders)

Please rate

Strongly disagree									Strongly Agree
1	2	3	4	5	6	7	8	9	10
☐	☐	☐	☐	☐	☐	☐	☐	☐	☐

2. I have the SOCIAL opportunity *to do enough physical activity for improvement to my health*

**What is SOCIAL opportunity?**<br><br>Interpersonal influences, social cues and cultural norms provide the opportunity to engage in the activity concerned<br><br>(e.g., support from friends and family)

Please rate

Strongly disagree									Strongly Agree
1	2	3	4	5	6	7	8	9	10
☐	☐	☐	☐	☐	☐	☐	☐	☐	☐

3. I am motivated to *do enough physical activity for improvement to my health*

**What is motivation?**<br>Conscious planning and evaluations (beliefs about what is good and bad)<br>(e.g., I have the desire to, I feel the need to)

Please rate

Strongly disagree									Strongly Agree
1	2	3	4	5	6	7	8	9	10
☐	☐	☐	☐	☐	☐	☐	☐	☐	☐

4. *Physical activity for improvement to my health* is something that I do automatically.

> **Automatic motivation** involves doing something without thinking or having to consciously remember
> (e.g., 'is something I do before I realise I'm doing it')

Please rate

Strongly disagree									Strongly Agree
1	2	3	4	5	6	7	8	9	10
☐	☐	☐	☐	☐	☐	☐	☐	☐	☐

5. I am PHYSICALLY able to *do enough physical activity for improvement to my health*.

> **What is PHYSICAL capability?**
>
> Having the physical skill, strength or stamina to engage in the activity concerned.
>
> (e.g., I have sufficient physical stamina, I can overcome disability, I have sufficient physical skills)

Please rate

Strongly disagree									Strongly Agree
1	2	3	4	5	6	7	8	9	10
☐	☐	☐	☐	☐	☐	☐	☐	☐	☐

6. I am PSYCHOLOGICALLY able to *do enough physical activity for improvement to my health*.

> **What is PSYCHOLOGICAL capability?**
>
> Knowledge and/or psychological skills, strength or stamina to engage in the necessary thought processes for the activity concerned.
>
> (e.g., having the knowledge, cognitive and interpersonal skills, having the ability to engage in appropriate memory, attention and decision making processes).

Please rate

Strongly disagree									Strongly Agree
1	2	3	4	5	6	7	8	9	10
☐	☐	☐	☐	☐	☐	☐	☐	☐	☐
